# Multi-attention fusion transformer for single-image super-resolution

**DOI:** 10.1038/s41598-024-60579-5

**Published:** 2024-05-03

**Authors:** Guanxing Li, Zhaotong Cui, Meng Li, Yu Han, Tianping Li

**Affiliations:** School of Physics and Electronics, Shandong Normal University, Jinan, Shandong China

**Keywords:** Super-resolution, Attention mechanism, Transformer, MAFT, Multi-attention fusion, Computer science, Information technology, Software

## Abstract

Recently, Transformer-based methods have gained prominence in image super-resolution (SR) tasks, addressing the challenge of long-range dependence through the incorporation of cross-layer connectivity and local attention mechanisms. However, the analysis of these networks using local attribution maps has revealed significant limitations in leveraging the spatial extent of input information. To unlock the inherent potential of Transformer in image SR, we propose the Multi-Attention Fusion Transformer (MAFT), a novel model designed to integrate multiple attention mechanisms with the objective of expanding the number and range of pixels activated during image reconstruction. This integration enhances the effective utilization of input information space. At the core of our model lies the Multi-attention Adaptive Integration Groups, which facilitate the transition from dense local attention to sparse global attention through the introduction of Local Attention Aggregation and Global Attention Aggregation blocks with alternating connections, effectively broadening the network's receptive field. The effectiveness of our proposed algorithm has been validated through comprehensive quantitative and qualitative evaluation experiments conducted on benchmark datasets. Compared to state-of-the-art methods (e.g. HAT), the proposed MAFT achieves 0.09 dB gains on Urban100 dataset for × 4 SR task while containing 32.55% and 38.01% fewer parameters and FLOPs, respectively.

## Introduction

The Single-Image Super-Resolution (SISR) aims to reconstruct a corresponding High-Resolution (HR) image using a Low-Resolution (LR) image. Early SR methods such as interpolation-based methods^1^, patch-based methods^2^, statistical-based methods^3,4^, and edge-based methods^5^ suffer from drawbacks like artifacts and missing texture details due to their lack of learning ability^6^. Notably, CNN-based models like SRCNN^7^, FSRCNN^8^, VDSR^9^, EDSR^10^, DRRN^11^, SRResNet^12^, and RCN^13^, can learn generalizable priors from the large dataset, and thus obtain reconstruction performance well above that of traditional methods. However, most CNN-based SR models resort to using small convolution kernels (e.g., 3 × 3), which limits the aggregation of input features and challenges the ability to provide extensive prior information for reconstruction tasks.

In recent years, the Transformer, which is based on attention mechanisms, has demonstrated effectiveness in capturing long-range dependencies and spatial correlations. As a result, it has gained widespread application in various computer vision tasks, including image classification, object detection, semantic segmentation, and super-resolution reconstruction. Nonetheless, the computational complexity of Transformer networks increases quadratically with the image size, resulting in a substantial computational burden when directly applied to image processing tasks. To address this issue, several models, including Twins^14^, SwinT^15^, Maxvit^16^, and CswinT^17^, have been developed to confine self-attention computations to the local windows. For example, SwinIR^18^, a modified version of Swin Transformer^15^, utilizes shifted windows to facilitate cross-regional interaction modeling, thereby mitigating the challenges associated with local distances and long-term spatial relationships. ELAN^19^ simplifies the architecture of SwinIR by using different window sizes and computing self-attention in larger windows to enhance the long-range modeling capability of the Transformer. NAT^20^ utilizes a simple sliding-window based Neighborhood Attention, localizes self-attention to the nearest neighbors around each token to enjoy a fixed attention span. CAT^21^ modifies the shape of the local windows and introduces rectangular window attention to achieve better reconstruction performance.

**Figure 1:**
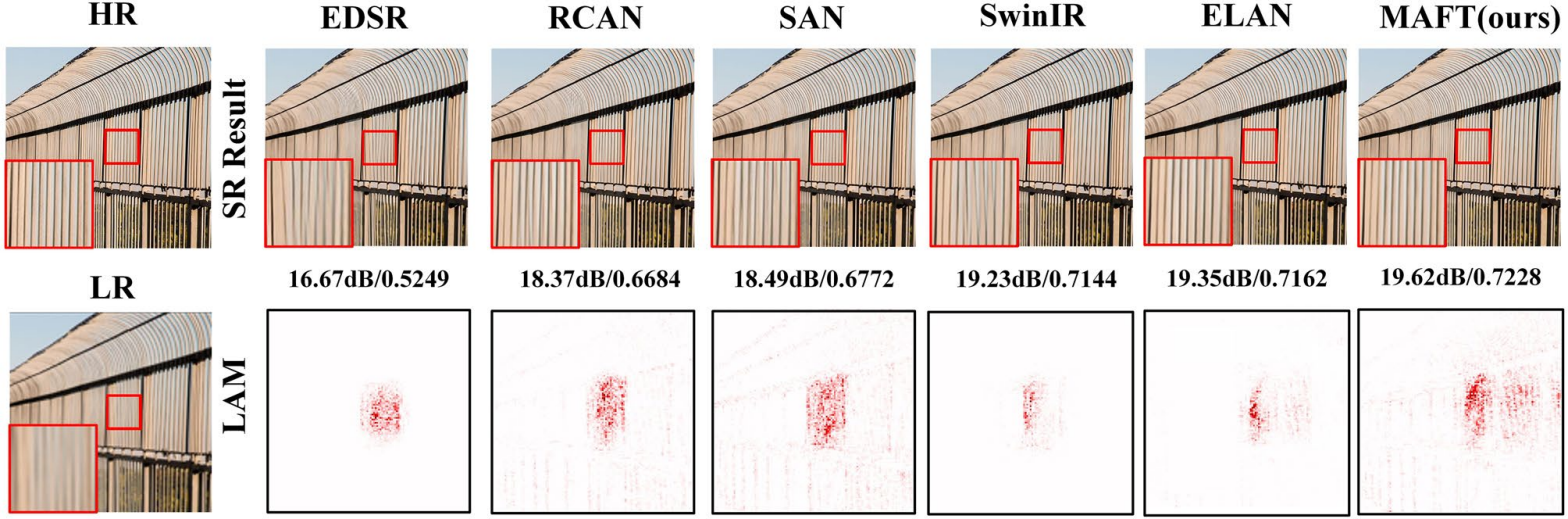
The results of the local attribution map (LAM) analysis for the CNN-based networks and transformer-based networks.

The Transformer-based networks described above achieved better performance than CNNs by modifying the local window. However, these networks are still limited by the local windows when performing the reconstruction task. To analyze pixel utilization differences among various network types during the reconstruction, we examine CNN-based SR networks, including EDSR^10^, RCAN^13^, and SAN^22^, as well as transformer-based networks such as SwinIR^18^ and ELAN^19^, using Local Attribution Maps (LAM^23^). The results are presented in Fig. 1. LAM is an attribution analysis method based on integrated gradients. It reflects which pixels in the input image contribute more to the reconstruction task. These pixels contain rich reconstruction information and are labelled as red dots on the image. By observing Fig. 1, it is evident that the number of highly contributing pixels labelled red in the LAM corresponding to SAN is significantly greater than that of EDSR and RCAN. And the quality of the image reconstructed with SAN is superior to that of the other two methods. This aligns with the general rule that higher pixel information results in better image reconstruction quality. However, when comparing the CNN-based SAN and Transformer-based SwinIR using the same strategy, we find that SwinIR utilizes significantly fewer pixels than SAN during the reconstruction, but the metrics (PSNR and SSIM) of the images reconstructed by SwinIR exceed those of SAN. This apparent contradiction to the general rule is actually due to the fact that Transformer-based models have more powerful mapping capabilities. They can complete the reconstruction task using less information. However, the Transformer-based network heavily relies on its powerful feature-mapping capability and utilizes a limited range of informative pixels for the reconstruction task. As a result, this often leads to the network incorrectly recovering texture details. For instance, when reconstructing the region highlighted by the red box in Fig. 1 using SwinIR and SAN, the former produced textures with noticeable errors compared to the HR image. In contrast, SAN can utilize more informative pixels and create texture similar to the HR image. This indicates that Transformer requires not only strong mapping capabilities, but also extensive pixel information to accurately reconstruct the details. Furthermore, upon observing the LAM results of SwinIR and ELAN, it is evident that ELAN activates a greater number of pixels than SwinIR during the reconstruction task. As a result, the quality of the reconstructed images produced by ELAN is superior to that of SwinIR. This demonstrates that enhancing the Transformer network's capability to activate pixels equally contributes to acquiring higher-quality reconstructed images.

Based on the above analyses, this paper aims to enhance the Transformer's ability to utilize input information, activate a wider range of pixels for image reconstruction, and improve network performance while ensuring accurate and reliable texture detail in the reconstructed image. Specifically, we propose a new Transformer network, named Multi-Attention Fusion Transformer (MAFT), for image super-resolution reconstruction tasks. In MAFT, we design a new attention module, Global Pixel Hybrid Attention (GPHA) module, which is inspired by Pixel Shuffle. By employing the shuffle operation in the global space, GPHA facilitates the spatial reorganization of global pixels within the feature map and enhances the connectivity among individual local windows. This approach broadens the scope of pixels utilized by the network, thereby increasing its overall effectiveness. To reduce computational load, GPHA adopts a method similar to^24^, shifting attention calculation from spatial to channel dimensions. Considering the limitations of GPHA in high-frequency feature extraction, we design the High-frequency Feature Enhanced (HFE) module. HFE extracts edge features with high-frequency information by introducing commonly used gradient operators in target detection tasks. The additional gradient operators are combined into a single deep convolution through the re-param operation^25^. This improves the network's performance without adding any computational cost. We replace the traditional Feed-Forward Network (FFN) with HFE, connected it separately to GPHA and Window-based Self-Attention (W-MSA), resulting in the Global Transformer Branch and Local Transformer Branch. To further enhance the ability of the Transformer network to utilize pixels, we have re-analyzed the LAM results presented in Fig. 1. Compared to RCNA and SAN, the EDSR network activates significantly fewer pixels in number and range. We consider that this is related to the channel attention that has been used in RCAN and SAN. Meanwhile, previous works^26–29^, have demonstrated that combining Transformer with CNN networks can significantly enhance network performance. Therefore, in MAFT, we introduce two CNN-based attention branches connected in parallel with the Transformer branches, which are Global CNN Attention Branch (GCAB) and Local CNN Attention Branch (LCAB). GCAB expands the receptive field of the network and activates a wider range of pixels by assigning different weights to each channel of the input feature, similar to channel attention. LCAB is responsible for extracting high-frequency information from input features to compensate for the Transformer branch's shortcomings in high-frequency feature extraction, resulting in improved visual performance.

This paper's main contributions can be summarized as follows:

1. We propose a new attention module, Global Pixel Hybrid Attention (GPHA), to spatially reorganize global pixel information in the feature map using the Shuffle operation, which effectively enhances the information interaction between different windows.
2. A High-frequency Feature Enhanced (HFE) module is designed to address the limitations of GPHA in high-frequency feature extraction. HFE enhances the network's high-frequency feature extraction capability without adding computational cost.
3. We combine Transformers with CNN-based attention branches in parallel to design a new SR model called Multi-Attention Fusion Transformer (MAFT). Extensive experiments on multiple datasets demonstrate that the proposed method MAFT could achieve comparable performance to the current state-of-the-art SR methods while using fewer parameters.

## Related work

In this section, we will briefly review related work, focusing on image super-resolution reconstruction technologies based on CNN, attention mechanisms, and the Transformer.

### SISR based on CNN

The richness of detailed information that can be obtained from an image is determined by its clarity, which in turn depends on the resolution. HR images typically contain significantly more detailed information compared to LR images. However, in practice, it is challenging to acquire HR images with the required texture details directly from natural sources. This challenge is closely associated with factors such as the image acquisition resolution of the camera, the size and type of the sensor, and the presence of noise during image processing^30^. LR images captured under the influence of various interference factors not only affect the visual sensory experience of individuals but also pose significant obstacles to tasks in computer vision, such as target classification and recognition. Consequently, enhancing image resolution has emerged as a critical objective within the discipline of image processing.

Due to the considerable cost investment required to improve the resolution of acquired images by enhancing the hardware performance of the image acquisition device. The production process significantly limits the feasibility of the hardware-based approach. Therefore, researchers have turned to software-based techniques as a more practical solution for increasing image resolution.

Single-image super-resolution reconstruction is a fundamental task in low-level vision, involving the recovery of a high-resolution image based on a given low-resolution image. CNN-based approaches have proven highly successful in single-image super-resolution reconstruction, thanks to their effective end-to-end feature representation capabilities. The initial CNN-based method introduced in this domain was SRCNN^7^, which utilized a three-layer convolutional network to achieve image reconstruction. Despite its shallow network depth, SRCNN's reconstruction quality surpassed that of traditional super-resolution methods such as bilinear interpolation^31^. Subsequent to SRCNN, an enhanced model known as FSRCNN^8^ was developed, significantly enhancing reconstruction speed and quality. This paved the way for widespread adoption of CNN-based techniques for image super-resolution reconstruction. VDSR^9^ pioneered the use of a 20-layer CNN for feature extraction, followed by EDSR^10^, which employed a deeper and broader CNN architecture with over 60 layers to extract richer and more detailed image features. RDN^32^ and RCAN^13^ further pushed the boundaries by utilizing CNN networks with over 100 and 400 layers, respectively, to perform super-resolution reconstruction tasks more effectively. Notably, increasing the number of CNN layers in the super-resolution task can substantially enhance model performance. However, as the network depth increases, so does the computational burden of the parameters, making model training more challenging. In response, Wang et al.^33^ proposed a lightweight and efficient super-resolution method, SMSR, to enhance real-time performance and enable integration into intelligent mobile devices. The success of CNNs in this context can be largely attributed to their inductive bias, allowing for more efficient and faster convergence through the exploitation of local attributes and weight sharing.

### SISR based on attention mechanism

The introduction of the attention mechanism has been effective in addressing the limitations of limited and fixed receptive fields in CNNs, particularly in long-distance dependent scenarios. For instance, Bengio et al.^34^ proposed an attention mechanism embedded in recurrent neural networks to explicitly establish a global dependency model over long distances by learning permutation and translation relationships between input and output sequences. The attention mechanism in neural networks can be viewed as a form of weighted average, and it encompasses primary attention mechanisms such as channel attention and spatial attention. For example, Hu et al.^35^ developed the SE network as a form of channel attention, while the CBAM method by Woo et al.^36^ integrates spatial attention and channel attention. Subsequently, various attention-based methods have emerged in image super-resolution reconstruction. For instance, Zhang et al.^13^ introduced the residual channel attention network (RCAN), which utilizes the attention module within the residual block to differentiate the features of different channels. Additionally, Dai et al.^22^ proposed the second-order channel attention module SOCAM and the second-order attention network (SAN) to address higher-order image features, building upon the first-order image feature approach of the SEnet by Hu. Moreover, Wei et al.^37^ identified varying reconstruction difficulties for different components (plane, edge, and diagonal) of the L1 loss function of EDSR, leading to the design of HGSR. Niu et al.^38^ modified the RCAN network to create the HAN network, which aggregates the output features of each residual block in the RCAN through layer attention blocks, in addition to adding an extra channel attention module at the end of each residual block. Furthermore, Qiao et al.^39^ proposed a Fourier domain attentional convolutional neural network and a Fourier domain attentional generative adversarial network model to investigate the characteristics of several image super-resolution reconstruction networks in the domain of microscopic image super-resolution, particularly with regard to video memory migration.

### SISR based on transformer

The introduction of the attention mechanism in the Sequence to Sequence model has significantly enhanced the reconstruction performance of the model. However, this improvement has been accompanied by a substantial increase in the difficulty of cross-sample parallel training, primarily due to memory limitations, leading to a considerable extension in the training time. To address this issue, the Transformer model, which consists of pure attention, was proposed and implemented in the field of machine translation. The versatile nature of the Transformer facilitates parallel training, thereby expediting the training process. Consequently, the Transformer has garnered increasing interest in the realm of Natural Language Processing (NLP), owing to its robust feature expression capability and structural diversity.

Some researchers, inspired by the field of NLP, have extended the Transformer model to the realm of computer vision. The pioneering work of DETR^40^ involved using a CNN backbone to extract features and implementing the Transformer structure to address target detection challenges. Subsequently, Dosovitskiy et al.^41^ applied the entire Transformer model to image classification, which resulted in an exceptional performance. This landmark development led to the emergence of various Transformer-based models that have dominated computer vision tasks like image super-resolution, classification, object detection, and semantic segmentation. Among these models, TTSR, developed by Fuzhi Yang et al.^42^, stands out as one of the earliest networks to leverage the Transformer architecture for image generation. Notably, TTSR facilitates the precise transfer of texture details from reference images to HR images through the integration of a texture converter with four closely linked modules. Moreover, subsequent to mastering the Swin Transformer, Liang et al.^18^ introduced SwinIR, a model specifically designed for image super-resolution reconstruction. SwinIR's shifted-window approach allows for the processing of large images without the need for patch division, thus enabling the network to restore high-frequency details, reduce blurring artifacts, and achieve significant reductions in computational costs.

## Methods

In this section, we will detail the Multi-Attention Fusion Transformer, referred to as the MAFT. We will first introduce the overall architecture of the MAFT, followed by the key modules Global Attention Aggregation (GAA) and Local Attention Aggregation (LAA) modules, respectively. We then give a detailed description of the different parts in the two modules.

### Multi-attention fusion transformer

**Figure 2:**
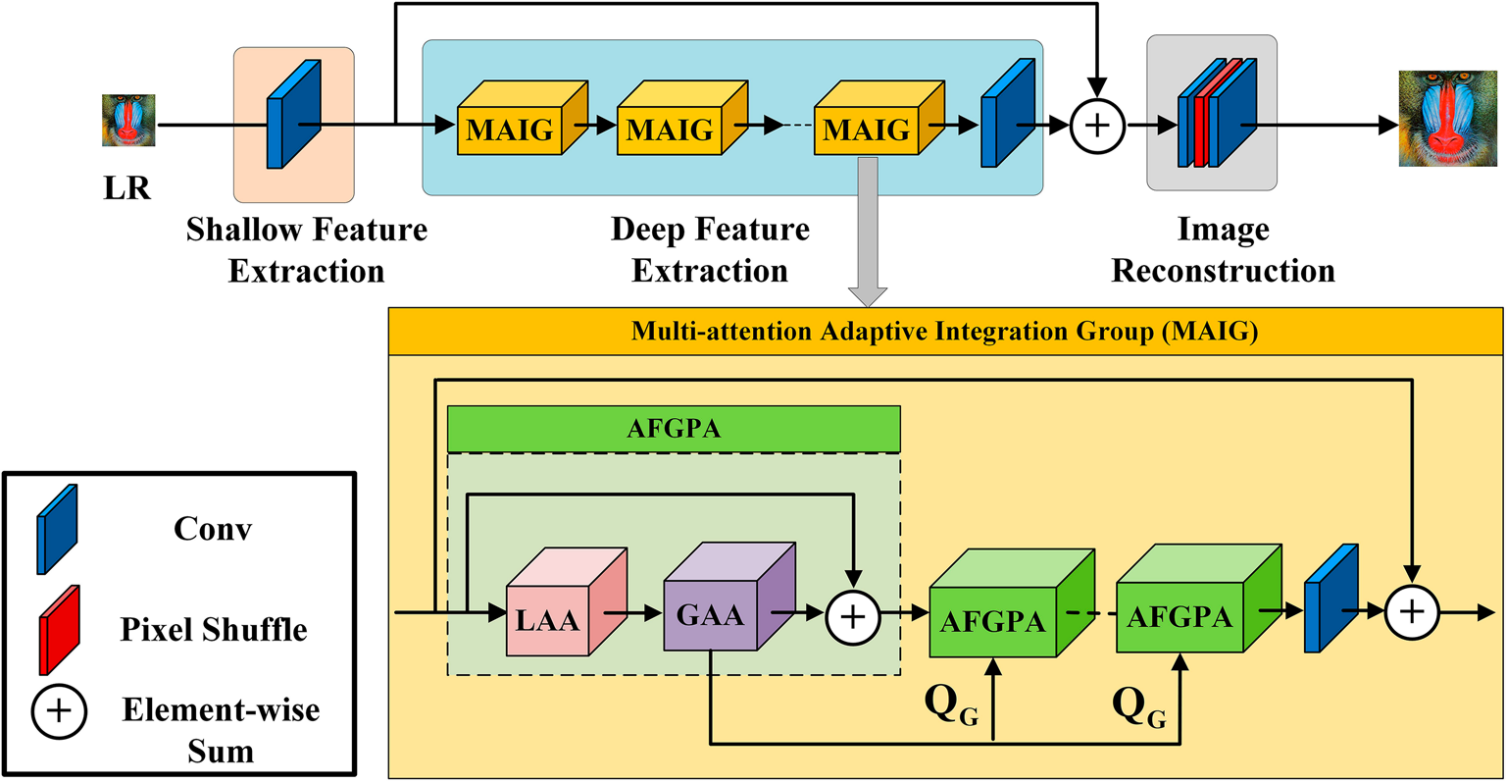
The overall structure diagram of the designed Multi-Attention Fusion Transformer (MAFT) and the Multi-attention Adaptive Integration Group (MAIG). MAFT uses single-layer convolution for shallow feature extraction, uses multiple cascaded MAIGs to explore in-depth features, and uses the strategy of pixel shuffle to upsample the fused features to obtain high-resolution images.

The overall architecture of the proposed MAFT is illustrated in Fig. 2. The network comprises three main components: shallow feature extraction, deep feature extraction, and image reconstruction. Given a low-resolution image ${I}_{LR}\in {\mathbb{R}}^{H\times W\times {C}_{in}}$, where $H$, $W$ and ${C}_{in}$ represent the height, width and channels of the input image, respectively. ${I}_{LR}$ first goes through a shallow feature extraction section, which initially extracts low-level image features in LR images and maps them into higher dimensions to obtain coarse features ${F}_{0}\in {\mathbb{R}}^{H\times W\times C}$. This process can be represented by the following equation:

1 $${F}_{0}={H}_{SF}({I}_{LR})$$

where $C$ denotes the channels of the intermediate features and $C\gg {C}_{in}$. ${H}_{SF}(\cdot )$ represents shallow feature extraction module and we use a $3\times 3$ convolution layer to achieve this function. By employing a simple convolution, low-level features such as edges, textures and fine-grained details can be effectively preserved, and ensures the high-dimensional embedding of each pixel label.

Next, the extracted shallow feature ${F}_{0}$ will be fed into the deep feature extraction block to further obtain depth features ${F}_{DF}\in {\mathbb{R}}^{H\times W\times C}$. This process can be described by the following equation:

2 $${F}_{DF}={H}_{{DF}_{MAIG}}({F}_{0})$$

where ${H}_{{DF}_{MAIG}}(\cdot )$ represents the deep feature extraction module composed of $N$ Multi-attention Adaptive Integration Groups (MAIGs) and a $3\times 3$ convolution layer. As shown in Fig. 2, each MAIG consists of $M$ sets of Alternating Fused Global Pixel Activation (AFGPA) modules and a $3\times 3$ convolution. A residual structure is employed to stabilize the training process. The intermediate processing can be expressed as follows:

3 $${F}_{i}={H}_{{conv}_{3\times 3}}\left({H}_{{AFGPA}_{M}}\left({F}_{i-1}\right)\right)+{F}_{i-1} i=\mathrm{1,2},\dots ,N$$

4 $${F}_{DF}={H}_{{conv}_{3\times 3}}({F}_{N})$$

where ${H}_{{conv}_{3\times 3}}(\cdot )$ represents a single-layer $3\times 3$ convolution which could better aggregate the in-depth feature information. ${F}_{i}$ represents the output features of $i{\prime}th$ MAIG module, and ${H}_{{AFGPA}_{M}}\left(\cdot \right)$ represents the $M$ stacked AFGPA modules. After successfully obtaining the depth feature ${F}_{DF}$, a global residual connection is used to combine the shallow and deep features. Subsequently, the reconstruction module reconstructs the SR image as the following formula:

5 $${I}_{SR}={H}_{Re}({F}_{0}+{F}_{DF})$$

where ${I}_{SR}$ represents the SR image obtained after reconstruction, and ${H}_{Re}(\cdot )$ represents the lightweight upsampling layer consisting of $3\times 3$ convolution and sub-pixel convolution layer.

### Alternating Fused Global Pixel Activation

The tremendous success of the Transformer in NLP can be attributed to its strategy of capturing contextual information by focusing on both distant and nearby tokens. However, the resulting vast quadratic computational complexity presents a major obstacle to its application in high-resolution image processing. SwinIR has sought to address this challenge by attempting to balance the dependence between short-term and long-term spatial information through the introduction of shifted windows, enabling the modeling of interactions across different regions. Nevertheless, the limited receptive field of local windows still greatly constrains the ability to capture distant information. The analysis of the local attribution map in introduction reveals that the shifted windows like those in SwinIR can cover only a small neighborhood around each window, the distribution of activated pixels remains dense. In contrast, the analysis of RCAN and SAN, which achieved a higher diffusion index in LAM, both demonstrates their ability to activate a more wider range of pixel values due to the introduction of channel attention in the network structure, involving the incorporation of global information in the computation process. Furthermore, the incorporation of convolution has been demonstrated to provides strong visual performance in many Transformer-based super-resolution models, adding significant value to network optimization (e.g.^26–29^, etc.).

**Figure 3:**
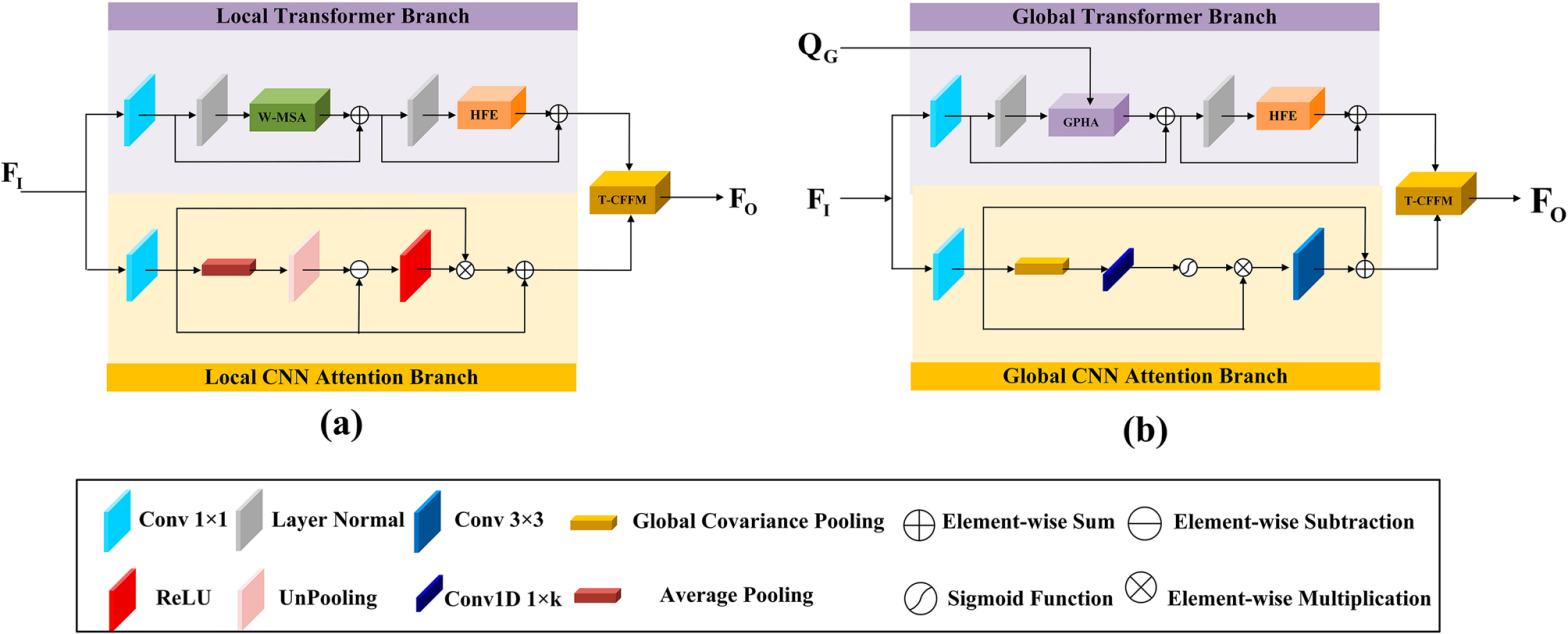
(**a**) The structure of the Local Attention Aggregation (LAA) module. (**b**) The structure of the Global Attention Aggregation (GAA) module.

After evaluating the network's performance and computational cost, we present the GAA and LAA modules to improve the balance between local features and global information, increase the number and distribution range of activated pixels, and ultimately improve the reconstruction performance of the network. The AFGPA module is formed by interconnecting these two modules alternately and employing a residual structure to enhance training stability. These two modules are shown in Fig. 3. Next, we will describe the GAA and LAA modules separately in detail.

### Global attention aggregation module

The architecture of the GAA module comprises two parallel branches, as illustrated in Fig. 3b. Taking the input feature ${F}_{GI}\in {\mathbb{R}}^{H\times W\times C}$ as an example, when the GAA receives this feature, it is first compressed by two $1\times 1$ convolutions. This process can be expressed as follows:

6 $${F}_{GTI}={H}_{{conv}_{1\times 1}}({ F}_{{\text{GI}}})$$

7 $${F}_{GCI}={H}_{{conv}_{1\times 1}}({ F}_{{\text{GI}}})$$

where ${F}_{GTI}\in {\mathbb{R}}^{H\times W\times \frac{C}{2}}$ and ${F}_{GCI}\in {\mathbb{R}}^{H\times W\times \frac{C}{2}}$ denote the input features of the Transformer branch and the CNN branch, respectively. ${H}_{{conv}_{1\times 1}}(\cdot )$ represents a $1\times 1$ convolution layer. By utilizing these two separate convolutions, the network can reduce the number of channels for intermediate feature mapping and consequently decrease the overall number of parameters. This reduction enhances both the training and inference efficiency of the network. ${F}_{GTI}$ and ${F}_{GCI}$ are then fed into the Global Transformer Branch and Global CNN Attention Branch respectively. The Global Transformer Branch leverages designed GPHA and HFE modules to extend attention computation to a global scale, enabling global dependency modeling. This enhances the network's ability to utilize more spatial distance information from LR images. The Global CNN Attention Branch improves the network's receptive field by generating channel-spatial attention maps, providing individual attention coefficients for each pixel. This ensures a wider range of pixel utilization by the network. Additionally, we introduce a Transformer-CNN Feature Fusion (T-CFF) module to merge the output features of two parallel branches. This approach prevents conflicts in visual optimization between the Transformer and CNN, while also utilizing the complementary strengths of both branches.

#### Global transformer branch

In the Global Transformer branch, ${F}_{GTI}$ first passes through the Layer Normalization layer to make feature distribution stable for attention training. Subsequently, we input the normalized features into the attention module GPHA. Besides, the query vector ${Q}_{G}$ generated by the Global Query Generator (GQG) module^43^, which contains the global contextual information, is also inputted into GPHA at the same time and participates in the computation of the global attention. To overcome the limitations of GPHA in extracting high frequency information, we develop the HFE module as a replacement for the conventional feed-forward network. In addition, we introduce the residual structure to avoid the effect of gradient explosion or gradient vanishing on network training. The introduction of residuals also allows deep features to retain more low-frequency information from shallow layers. The calculation process of the Global Transformer Branch is as follows:

8 $${F}_{GPHA}={H}_{GPHA}(LN\left({F}_{GTI}\right), {Q}_{G})$$

9 $${F}_{GTO}={H}_{HFE}(LN({F}_{GPHA}+{F}_{GTI}))+{F}_{GPHA}$$

where ${F}_{GPHA}$ and ${F}_{GTO}$ denote the depth global features output by the GPHA module and the final output features of the Global Transformer branch, respectively, ${H}_{GPHA}(\cdot )$ and ${H}_{HFE}(\cdot )$ represent the GPHA and HFE modules we designed, respectively. Detailed explanations will be provided in subsequent chapters.

#### Global pixel hybrid attention

Before formally introducing the GPHA module designed by us, we first analyze the standard self-attention computation process. Take input feature ${F}_{I}\in {\mathbb{R}}^{H\times W\times C}$ as an example, where $H$, $W$ and $C$ denote the height, width and channels of the input feature respectively. ${F}_{I}$ is first expanded by the one-dimensional into $X\in {\mathbb{R}}^{N\times C}$, where $N=H\times W$. Next, matrices ${W}^{Q}$, ${W}^{K}$, and ${W}^{V}$ respectively map $X$ to three matrices: query $Q\in {\mathbb{R}}^{N\times D}$, key $K\in {\mathbb{R}}^{N\times D}$, and value $V\in {\mathbb{R}}^{N\times D}$, where $D$ represent the number of channels. In general, $D\gg C$. The increase in channel dimension allows the network to capture richer feature information, but also increases the computational cost during training and inference. Next, the network performs a processing transformation on the query $Q$ and the key $K$ to obtain an attention graph containing all the input relevance information, which is used in a weighted sum of value $V$ to finally obtain the attention output. The complete attention computation process can be formulated as follows:

10 $$Q=X{W}^{Q}, K=X{W}^{K}, V=X{W}^{V}$$

11 $$Attention\left(Q,K,V\right)=\phi (Q,K)V$$

where $\phi (Q,K)$ represents the attention map that includes relevant information, which is typically obtained by applying the dot-product attention calculation to $Q$ and $K$, and processing the similarity matrix using the softmax function. The attention calculation formula at this point is as follow:

12 $$Attention\left(Q,K,V\right)=softmax(\frac{Q{K}^{T}}{\sqrt{D}})V$$

Currently, standard self-attention has a computational complexity of $O({N}^{2}D+N{D}^{2})$. Since $N\gg D$, it can be approximated that the computational cost grows quadratically with the input resolution. Obviously, the computational cost of computing attention directly on the input image is enormous, so strategies such as W-MSA attempts to confine the computation of attention to a localized window. Specifically, the input features are divided into $\frac{HW}{{M}^{2}}$ non-overlapping local windows of size $M \times M$, and the computation of attention is performed individually within each local window. This processing greatly reduces the computational cost, but also limits the ability of the network to model long-range pixel dependencies. Although SwinIR tries to improve the connection between different local windows by introducing shifted windows, it can be seen from the previous LAM analysis that this shifted window strategy still makes it difficult to use the pixel information in a wider range, and the reconstructed image is still prone to errors in texture details.

**Figure 4:**
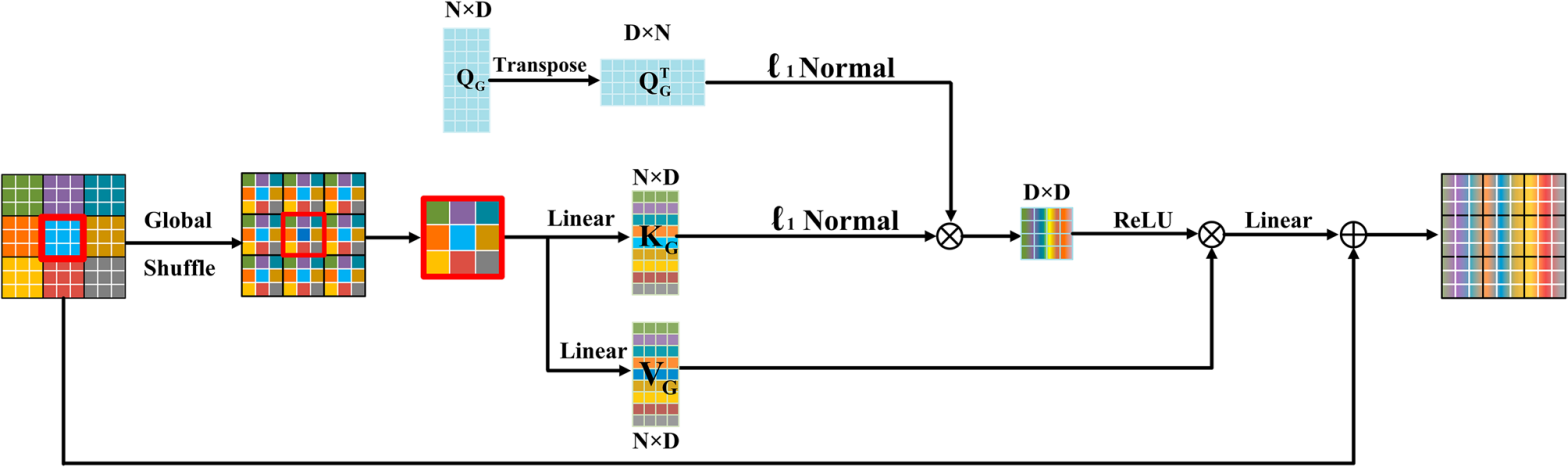
The schematic diagram of the global pixel hybrid attention (GPHA).

To enhance the network's capability in utilizing spatial range information from input images, broaden the range of activated pixels during the reconstruction task, and minimize computation costs, we propose the GPHA, which is illustrated in Fig. 4. For clarity, Fig. 4 displays the processing of input features on a single channel.

**Figure 5:**
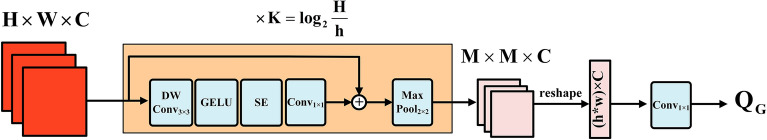
The structure of the global query generator (GQG) module.

When the GPHA module receives the input feature ${F}_{GTI}\in {\mathbb{R}}^{H\times W\times \frac{C}{2}}$, the network first uses the Global Query Generator module^43^ to extract the query vector which contains the global context information in the global perspective. The structure of this module is shown in Fig. 5. The GQG consists of $K$ (${\text{K}}={{\text{log}}}_{2}\frac{H}{M}$) Fused MBConv and $K$ Average Pooling alternately connected. Each Fused MBConv contains a $3\times 3$ convolution, GELU activation function, squeeze excitation module, $1\times 1$ convolution, and residual structure. Fused MBConv can extract desirable properties such as inductive bias and modeling of inter-channel dependencies. Average Pooling makes sure that the global features extracted end up being the same size as the local window. After reshaping the extracted global features, a $1\times 1$ convolution is required to expand their channel number from $\frac{C}{2}$ to $D$ to match the dimensions of the subsequent extracted global keys and values. The complete process of extracting the global query ${Q}_{G}$ can be represented by the following formulas:

13 $${x}_{i}=AvgPool\left({H}_{{conv}_{1\times 1}}\left(SE\left(GELU\left({H}_{{conv}_{3\times 3}}\left({x}_{i-1}\right)\right)\right)\right){+x}_{i-1}\right) i=\mathrm{1,2},3\dots {\text{K}}$$

14 $${Q}_{G}={H}_{{conv}_{1\times 1}}({R}_{S}({x}_{K}))$$

where ${x}_{i}$ represents the output after processing by the $ith$ Fused MBConv and Average Pooling, $GELU(\cdot )$ represents the Gaussian Error Linear Units, $SE(\cdot )$ represents the squeeze excitation module, ${R}_{S}(\cdot )$ represents the Reshape processing. Note that in the MAFT network we designed, the GTG module only needs to participate in the computation in the first AFGPA module in each MAIG, and the generated global query tokens can be directly applied to the other AFGPA modules in the current MAIG, which is advantageous for saving computational costs. This is one of the important reasons why we use GQG to generate the global query.

To further expand the pixel activation range, inspired by the Pixel Shuffle operation, we consider $\frac{HW}{{M}^{2}}$ non-overlapping local windows with size $M\times M$ as the input features, which have the dimension with $M\times M\times \frac{HW}{{M}^{2}}\frac{C}{2}$. By applying spatial shuffle to these windows, we spatially reorganize the global pixel information of feature maps, enhancing the connections between the individual local windows. Next, we re-divide the shuffled feature map into non-overlapping local windows of size $M\times M$. Each local window now contains abundant global pixel information. Following the standard self-attention approach, we calculate global key matrix ${K}_{G}\in {\mathbb{R}}^{N\times D}$ and value matrix ${{\text{V}}}_{G}\in {\mathbb{R}}^{N\times D}$ within each local window.

When computing the attention graph, GPHA does not use the approach in Eq. (12). On the one hand, performing the dot product computation directly on the query and key matrices in the spatial dimension would add $O({N}^{2}D)$ computational complexity and increase the training burden for the network. On the other hand, the softmax function assigns non-zero attention weights to all given context elements based on the relevance information obtained from the dot product, even if some of these elements are irrelevant to the query or contain noise. This dense attention mechanism drags down the network reconstruction performance while increasing the amount of redundant computation. And in our proposed GPHA, due to the global pixel mixing strategy, this detrimental effect will be more significant. Inspired by^24^, we shift the computation of the attention map to the channel dimensions to obtain global dependencies. Specifically, we perform the transpose operation on the extracted global query ${Q}_{G}$ to obtain ${Q}_{G}^{T}\in {\mathbb{R}}^{D\times N}$. We then perform normalization operations on each channel of the transposed global query and global key, respectively, which can be expressed as follows:

15 $${\widehat{Q}}_{G}^{Ti}=\frac{{Q}_{G}^{Ti}}{{\Vert {Q}_{G}^{Ti}\Vert }_{1}}$$

16 $${\widehat{K}}_{G}^{i}=\frac{{K}_{G}^{i}}{{\Vert {K}_{G}^{i}\Vert }_{1}}$$

where ${Q}_{G}^{Ti}$ and ${K}_{G}^{i}$ represent the $ith$ row of ${Q}^{T}$ and $ith$ column of $K$, respectively. ${\widehat{Q}}_{G}^{Ti}$ represents the results of ${Q}_{G}^{Ti}$ normalzation, while ${\widehat{K}}_{G}^{i}$ represents the results of ${K}_{G}^{i}$ normalization. ${\Vert \cdot \Vert }_{1}$ stands for ${\mathcal{l}}_{1}$ normalization. This normalization strategy, which is an effective alternative to the softmax function, ensures the normalization of attention. After obtaining the normalized transposed query matrix ${\widehat{Q}}_{G}^{T}$ and the normalized key matrix ${\widehat{K}}_{G}$, we calculate the dot product between the them to obtain the attention graph with similarity information, and the formula is as follow:

17 $$\phi \left(Q,K\right)={\widehat{Q}}_{G}^{T}{\widehat{K}}_{G}$$

The computational complexity of the attention mechanism has changed from ${\text{O}}({N}^{2}{\text{D}})$ to ${\text{O}}({\text{N}}{D}^{2})$. Considering $D\ll N$, this significantly reduces the computational cost and improves the reconstruction efficiency of the network. However the attention map generated using the normalized dot product operation still assigns non-zero weights to all contextual elements, and in some cases the weights of certain elements may become negative due to significant differences between the query and key vectors. To eliminate the influence of irrelevant elements on the attention map, inspired by sparse attention, we introduce a $ReLU$ activation after the dot product. $ReLU$ retains only the positive correlations and sets the weights of irrelevant or noisy elements to zero. In this way, we obtain a sparse attention map to ensure that the model focuses on processing highly correlated elements. In addition, to ensure stability during network training, we multiply a learnable scaling parameter $\frac{1}{\rho }$ before the $ReLU$ function to adaptively adjust the value of the attention graph. The full GPHA attention formula is shown below:

18 $${Attention}_{GPHA}\left({\text{Q}},{\text{K}},{\text{V}}\right)=\frac{{V}_{G}\cdot ReLU({\widehat{Q}}_{G}^{T}{\widehat{K}}_{G})}{\rho }$$

#### High-frequency feature enhanced module

**Figure 6:**
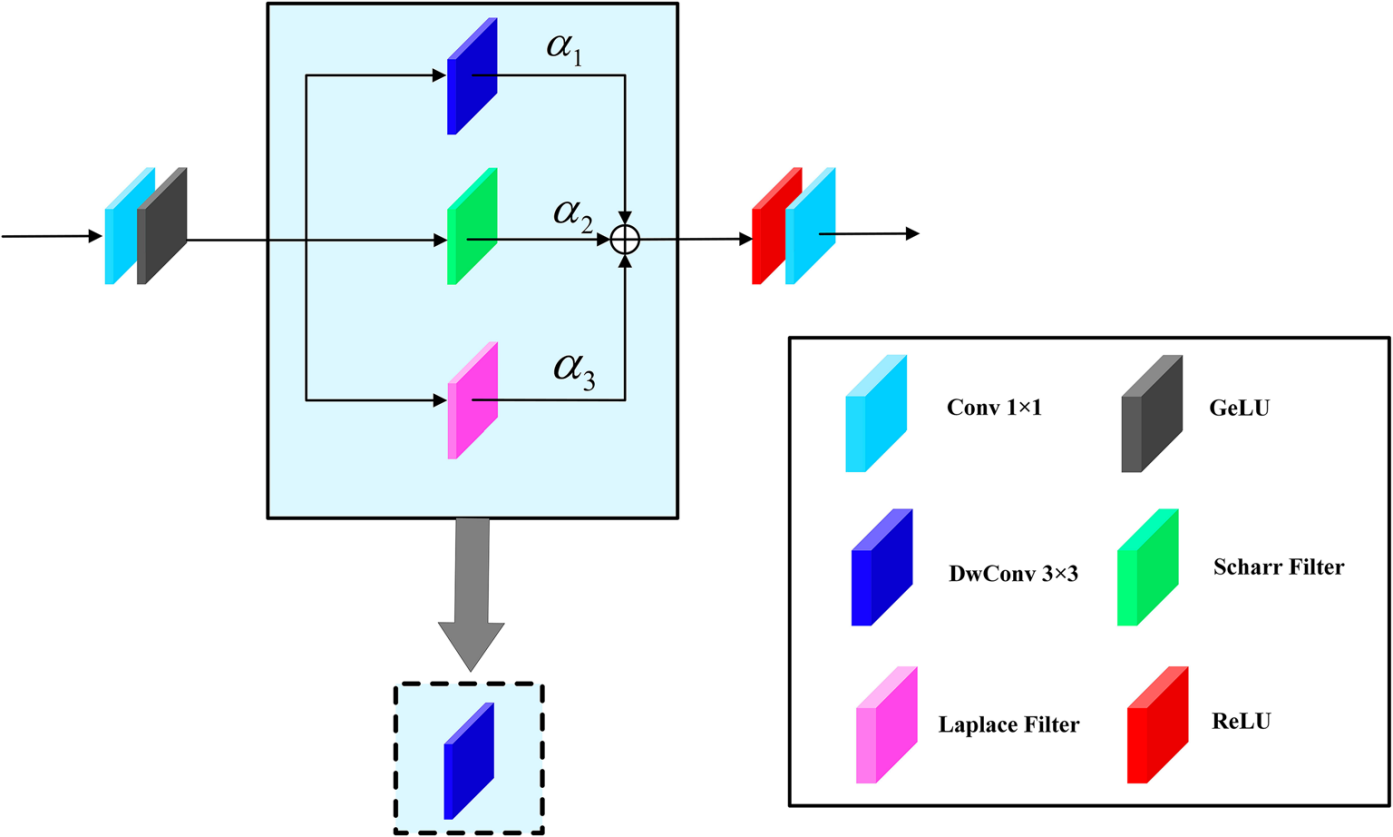
The structure of the High-frequency Feature Enhanced (HFE) module.

Our proposed GPHA is a sparse attention mechanism that primarily focuses on low-frequency information at a global scale, while neglecting high-frequency information such as sharp edges required for image texture reconstruction. To address this issue, we introduce the parallel CNN branches in the network, which will be better at capturing local high-frequency information. Additionally, we integrate the edge detection operator into the vanilla FFN to enhance the ability of the Transformer branch in extracting high-frequency information. We named the modified FFN as High-frequency Feature Enhanced module. Inspired by^25^, we simplified the parallel high-frequency feature extraction branches during the inference stage by re-parameterizing them into a single depth-wise convolution (DwConv) layer. This ensures that HFE does not introduce extra computational complexity. The specific structure of HFE is shown in Fig. 6.

The input features of HFE are processed through a $1\times 1$ convolution and GELU activation function for dimension reduction. Subsequently, the reduced features are fed in parallel to three branches for high-frequency information extraction, denoted as ${F}_{i}$ for each branch. The top branch consists of a $3\times 3$ DwConv. We use ${K}_{DwConv}$ and ${B}_{DwConv}$ denote the learnable kernel weights and bias of this DwConv, respectively, use ∗ to represent the convolution operation. The feature extraction process can be represented by the following formula:

19 $${F}_{DwConv}={K}_{DwConv}*{F}_{i}+{B}_{DwConv}$$

In the intermediate branch, we incorporate the Scharr filter commonly used for image edge detection. This operator, serving as a 1st-order gradient convolution kernel, exhibits strong edge responses, effectively capturing the high-frequency information required by the network. It contains two $3\times 3$ convolution kernels, used to compute the image gradients in the horizontal and vertical directions, labeled as ${K}_{{Sch}_{X}}$ and ${K}_{{Sch}_{Y}}$, respectively. After expanding and repeating the two convolutional kernels, we use them to extract gradient information for feature extraction. This process can be represented as follows:

20 $${F}_{Scharr}={K}_{{Sch}_{X}}*{F}_{i}+{B}_{{Sch}_{X}}+{K}_{{Sch}_{Y}}*{F}_{i}+{B}_{{Sch}_{Y}}$$

where ${B}_{{Sch}_{X}}$ and ${B}_{{Sch}_{Y}}$ denote the bias of the Scharr filter in the horizontal and vertical directions, respectively. A commonly used second-order gradient operator, Laplace filter, is introduced in the bottom branch. Specifically, we employed $3\times 3$ Laplace filters with 4-neighborhood and 8-neighborhood, denoted as ${K}_{{L}_{4}}$ and ${K}_{{L}_{8}}$, respectively. The corresponding biases are labeled as ${B}_{{L}_{4}}$ and ${B}_{{L}_{8}}$. The process of extracting second-order gradient information using Laplace filters can be represented by the following formula:

21 $${F}_{Laplace}={K}_{{L}_{4}}*{F}_{i}+{B}_{{L}_{4}}+{K}_{{L}_{8}}*{F}_{i}+{B}_{{L}_{8}}$$

Finally, we individually weight the features output by these three branches, resulting in high-frequency features represented as follows:

22 $${F}_{HF}={\alpha }_{1}{F}_{DwConv}+{\alpha }_{2}{F}_{Scharr}+{\alpha }_{3}{F}_{Laplace}$$

where ${\alpha }_{1}$, ${\alpha }_{2}$ and ${\alpha }_{3}$ are the learnable parameters. Following the method proposed in^25^, we re-parameterize the three branches, and denote the combined kernel weights and bias as $K$ and $B$, respectively. These parameters can be obtained by the following equation:

23 $$K={\alpha }_{1}{K}_{DwConv}+{\alpha }_{2}({K}_{{Sch}_{X}}+{K}_{{Sch}_{Y}})+{\alpha }_{3}({K}_{{L}_{4}}+{K}_{{L}_{8}})$$

24 $$B={\alpha }_{1}{B}_{DwConv}+{\alpha }_{2}({B}_{{Sch}_{X}}+{B}_{{Sch}_{Y}})+{\alpha }_{3}({B}_{{L}_{4}}+{B}_{{L}_{8}})$$

The above operation allows the three parallel branches of high-frequency feature extraction to be merged into a single DwConv layer, improving the network's ability to extract high-frequency information without introducing additional computational complexity. The extracted high frequency features can be represented as follows:

25 $${F}_{HF}=K*{F}_{i}+B$$

Finally, like traditional FFNs, by passing ${F}_{HF}$ through the $ReLU$ function and a $1x1$ convolution, we will obtain the final output of HFE module.

#### Global CNN attention branch

Previous research^26–29^ has shown that combining CNN and Transformer can significantly enhance network performance due to their respective strengths. In GAA, we also introduce a CNN-based Global Attention Branch which is parallel to the Global Transformer Branch. This branch helps to expand the receptive field of the network by distinguishing between different image patches in each channel and assigning weights to different channels. This allows a wider range of pixel information to be activated. The structure of this CNN branch is illustrated in Fig. 3b. Given an input feature ${F}_{GCI}\in {\mathbb{R}}^{H\times W\times \frac{C}{2}}$, the feature is first processed through a global covariance pooling layer to obtain a 3D tensor ${F}_{GCP}\in {\mathbb{R}}^{1\times 1\times \frac{C}{2}}$, which is rich in inter-channel correlation information. To balance network performance and computational complexity while exploring nonlinear interactions between channels, we adopt the strategy in^44^, input ${F}_{GCP}$ into a one-dimensional convolution of size $k$ to facilitate information exchange between channels. The size of the one-dimensional convolutional kernel is adaptively calculated by the following formula:

26 $$k=\left|\frac{{log}_{2}(\frac{C}{2})}{\gamma }+\frac{b}{\gamma }\right|$$

where $\gamma$ and $b$ are used to control the degree of cross-channel interaction, typically set as $\gamma =2$ and $b=1$. Next, we apply the sigmoid function to the one-dimensional convolution output to obtain a channel attention mask that captures inter-channel relationships. By multiplying it with the input feature ${F}_{GCI}$ and aggregating the features through a $3x3$ convolution, we obtain an attention map of dimension ${\mathbb{R}}^{H\times W\times \frac{C}{2}}$, where each pixel has its own attention coefficient. Finally, we stabilize the training process by connecting the attention map with the input feature map ${F}_{GCI}$ using a residual structure. The attention computation process described above can be represented by the following formula:

27 $${F}_{GCO}={H}_{{Conv}_{3\times 3}}\left[{F}_{GCI}\otimes \sigma ({H}_{{Conv}_{k}}(GCP({F}_{GCI})))\right]+{F}_{GCI}$$

where $GCP(\cdot )$ denotes the global covariance pooling layer, ${H}_{{Conv}_{k}}(\cdot )$ and ${H}_{{Conv}_{3\times 3}}(\cdot )$ denote the 1D convolution with kernel size $k$ and the 2D convolution with kernel size $3 \times 3$, respectively, $\sigma$ denotes the sigmoid function, ⊗ denotes the element-by-element multiplication, and ${F}_{GCO}$ denotes the output features of the Global CNN Attention branch in GAA.

#### Transformer-CNN feature fusion module

**Figure 7:**
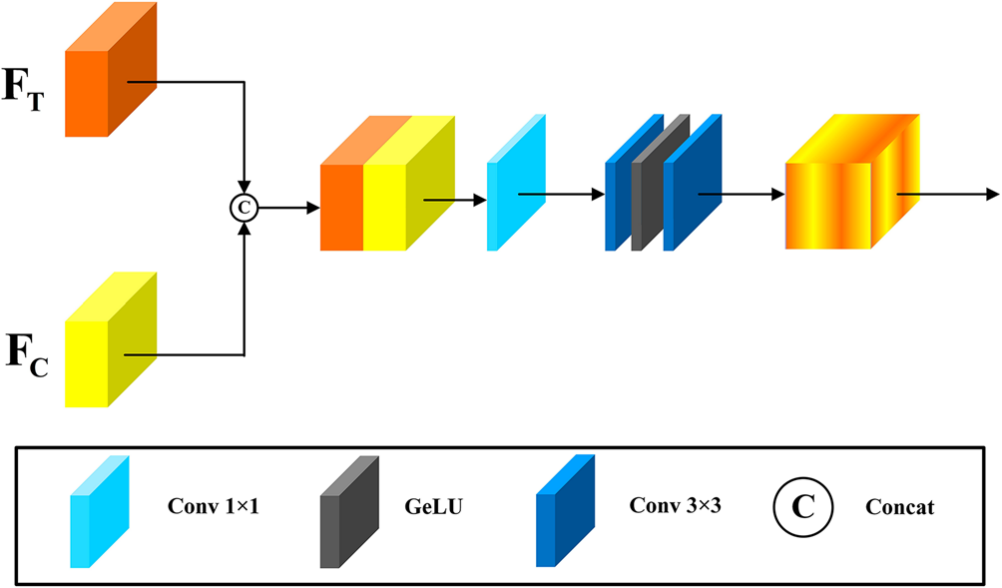
The structure of the transformer-CNN feature fusion (T-CFF) module.

After extracting output features ${F}_{GTO}$ from the Transformer branch and output features ${F}_{GCO}$ from the CNN branch, we need to merge these features and the advantages of the two branches will be complementary. In this paper, the structure of the T-CFF module we design is shown in Fig. 7.

We first perform concatenation operations between ${F}_{GTO}$ and ${F}_{GCO}$ in the channel dimension to get the merged feature ${F}_{TC}\in {\mathbb{R}}^{H\times W\times C}$. Subsequently, a $1\times 1$ convolutional layer is used to fuse features along the channel dimension. Finally, two $3\times 3$ convolutions and a GELU activation function are used to improve the network's ability to extract local neighborhood information. The complete feature fusion process can be represented by the following formulas:

28 $${F}_{Fusion}={H}_{{Conv}_{1\times 1}}[{H}_{C}({F}_{GTO},{F}_{GCO})]$$

29 $${F}_{GO}={H}_{{Conv}_{3\times 3}}(GELU({H}_{{Conv}_{3\times 3}}({F}_{Fusion})))$$

where ${H}_{C}(\cdot )$ denote the concatenation operation, ${F}_{Fusion}$ and ${F}_{GO}$ denote the fusion features and the final output features of the GAA module, respectively.

### Local attention aggregation module

In LAA, we also adopt the dual parallel branch structure, as shown in Fig. 3a. Similar to the GAA, the input features are first compressed by two 1 × 1 convolution before extracting deep features in both the Local Transformer branch and the Local CNN Attention branch. In the Local Transformer Branch, we apply the classical W-MSA directly to calculate attention in the local windows. We also replace the traditional FFN with the designed HFE module which does not introduce additional computational complexity.

In the CNN branch, we integrated the Local CNN Attention Branch to further extract high-frequency information from the features. As shown in Fig. 3a, given the input feature ${F}_{LCI}\in {\mathbb{R}}^{H\times W\times \frac{C}{2}}$, we calculate the average within each patch through the average pooling, resulting in a pooled feature map which characterizes the average strength of each patch. Subsequently, we perform unpooling on the pooled feature map to obtain the low-frequency feature map which has the same dimension as the input features. To highlight pixels with high values, we subtract the low-frequency feature map from the input feature and apply the ReLU activation function. We then element-wise multiply the result with the input feature to preserve and enhance high-frequency information above the mean, while discarding low-frequency information below the mean. Additionally, we introduce a residual structure to stabilize the training process. These operations can be represented by the following formulas:

30 $${F}_{H}=ReLU[{F}_{LCI}-{H}_{Unpool}({H}_{Avg}({F}_{LCI}))]$$

31 $${F}_{LCO}={F}_{LCI}\otimes {F}_{H}+{F}_{LCI}$$

where ${H}_{Avg}(\cdot )$ and ${H}_{Unpool}(\cdot )$ denote the average pooling operation and the unpooling operation, respectively, ${F}_{H}$ and ${F}_{LCO}$ denote the residual feature maps enriched with high-frequency information and the outputs of the Local CNN Attention Branche, respectively. After obtaining the outputs from the Local Transformer branch and Local CNN Attention branch separately, they are inputted into T-CFFM for feature fusion following the same computational process as Eqs. (28) and (29).

## Experiments

In this section, we first introduce the details of datasets, evaluation metrics and implementation details. We verified the effectiveness of the modules in the MAFT network through ablation experiments, and finally quantitatively and qualitatively compared the reconstruction results of our designed MAFT with the state-of-the-art networks on five benchmark datasets.

### Datasets and evaluation metrics

We use DF2K, a high-quality dataset formed by DIV2K^45^ and Flickr2K^46^ datasets, as our training dataset. DF2K is widely used in image super-resolution reconstruction, and the LR images are obtained by bicubic degradation of the corresponding HR images. We carry out experiments under upscaling factors: × 2, × 3 and × 4 and use five commonly available benchmark datasets, including Set5^47^, Set14^48^, BSD100^49^, Urban100^50^, and Mangan109^51^ as test datasets to compare model performance and generalization ability. All of the above datasets are generic and can be accessed at the address given in the cited literature. PSNR^52^ and SSIM^53^ are used to judge the quality of the reconstructed images. We visualize the distribution of activated pixels in the reconstruction task by local attribution map^23^.

### Implementation details

The specific module parameters in the MAFT network are set as follows: The number of MAIG is set to 6 and the number of AFGPA modules in each MAIG module is set to 2. Since there are two modules LAA and GAA in each AFGPA, the total number of Transformer blocks in MAFT is 24. The number of attention heads in both W-MSA and GPHA is set to 6, and the window size is set to 16. The channel number of the whole network is set to 180. During the training, a mini-batch consists of eight images of size $64\times 64$, randomly cropped from the training dataset, and data augmentation is performed by random rotations and horizontal flips of ${90}^{^\circ }$, ${180}^{^\circ }$ and ${270}^{^\circ }$. The network parameters are optimized by the ${L}_{1}$ function. We use ADAM optimizer to optimize the network with parameter set as: ${\beta }_{1}$=0.9, ${\beta }_{2}=0.999$, $\epsilon ={10}^{-8}$. The initial learning rate is set to ${10}^{-4}$ and will be half at milestones: [250 K,400 K,450 K,475 K]. We implement the model using Pytorch, and all experiments were carried out in GTX 3090 GPUs.

### Ablation experiment

This section presents several ablation experiments to validate the effects of the various components designed in the MAFT on the reconstruction results. For comparison, we design a baseline model A. This baseline model replaces all GPHAs in our designed MAFT with W-MSAs and replaces our designed HFEs with the standard FFNs, while removing all CNN branches. The baseline network and all subsequent networks in this section share the same implementation details (e.g. the same channels and attention heads). They are trained on the DF2K dataset and evaluated on the Urban100^50^ dataset. Considering the training cost, the number of iterations is set to 300 K during the ablation experiments.

#### Effectiveness of GPHA and HFEM

**Figure 8:**
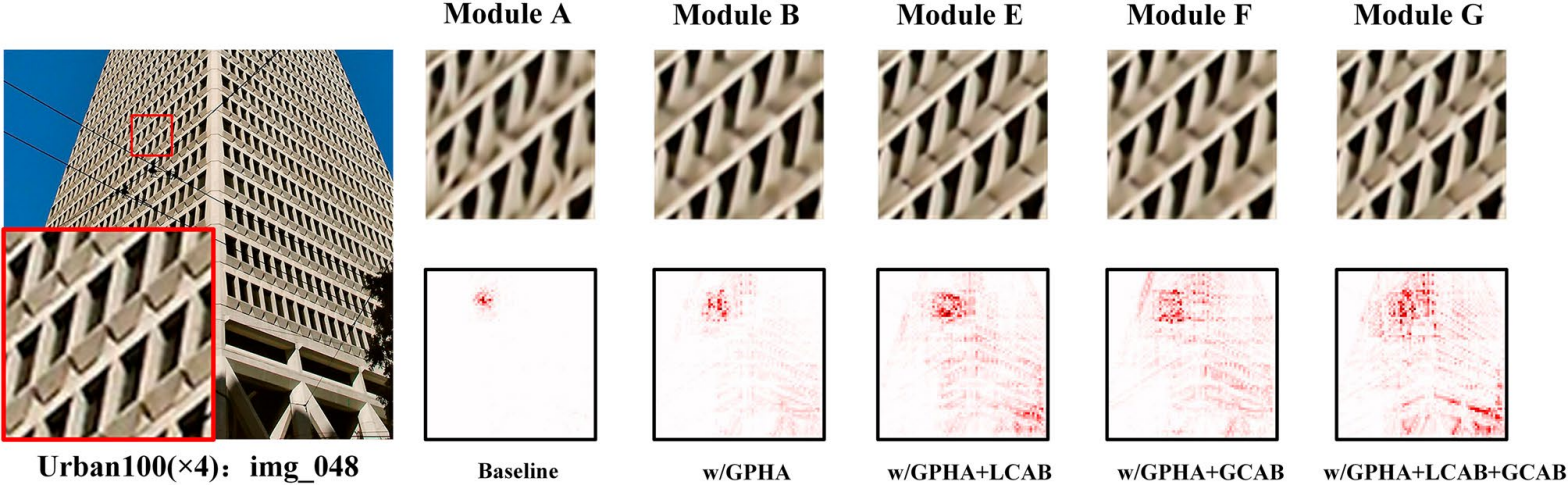
The outcomes of LAM analysis for various models.

Table 1 shows the effectiveness of GPHA and HFE. Three additional networks are designed alongside the baseline model. Keeping the total number of Transformer blocks unchanged, we replaced half of the W-MSAs in the baseline model with GPHAs to obtain the model B, which consists of alternately connected W-MSAs and GPHAs. Comparing the reconstruction results of model B with the baseline model A on the Urban100 dataset $\times 4$ scale factor, it can be observed that the introduction of GPHA improves the PSNR and SSIM of the reconstructed images by 0.11 dB and 0.002, respectively. However, the resulting increase in the number of parameters is only 0.29 M. Since GPHA was originally designed to expand the number and range of pixels activated by the network during reconstruction, we analyze the pixel utilization in reconstruction for the baseline model A and model B separately using the local attribution map method which can represent the range of the attributed pixels, and the results are shown in Fig. 8. It is clear that model B with GPHA has a larger range of utilized pixels compared to the baseline model, which is attributed to the global attention strategy used in GPHA.

**Table 1:** Ablation studies on GPHA and HFE, the metrics PSNR and SSIM are calculated on the Urban100 dataset with a scaling factor of 4.

Model	Model A	Model B	Model C	Model D
GPHA	$\times$	$\surd$	$\times$	$\surd$
HFE	$\times$	$\times$	$\surd$	$\surd$
PSNR/SSIM	27.69/0.8331	**27.80/0.8351**	27.74/0.8345	*27.89/0.8359*
Parameters(M)	9.58	9.87	9.87	9.87

The best and second best results are marked in italics and bold, respectively.

By replacing the FFN network in the baseline model with the designed HFE, model C is obtained. Compared to the baseline model, the introduction of the HFE results in an improvement of 0.05 dB and 0.0014 in the PSNR and SSIM of the reconstructed image, respectively. This demonstrates the effectiveness of HFE in compensating for the Transformer’s ability to extract high-frequency information. Additionally, the use of re-parameterization ensures that HFE does not impose additional computational complexity during the inference phase. It is worth mentioning that the performance improvement achieved by combining HFE with GPHA (model D) is more pronounced than that achieved by combining HFE with W-MSA (model C). Using PSNR as an example, when comparing models A and C, the introduction of HFE resulted in a performance improvement of 0.05 dB. However, when comparing models B and D, the introduction of HFE resulted in an improvement of 0.09 dB. This suggests that GPHA is more concerned with extracting low-frequency information than W-MSA. Therefore, high-frequency feature extraction module is crucial to GPHA.

#### Effectiveness of CNN branch

To highlight the importance of incorporating CNN branches in the Transformer network, we chose model B in Table 1 as a new baseline model to perform ablation experiments on LCAB and GCAB branches, respectively. Since both HFE and LCAB aim to address the Transformer branch’s limitations in extracting high-frequency features, in order to better demonstrate the effect of LCAB on the Transformer branch separately, we did not select model C or D in Table 1 as the new baseline model. The results are presented in Table 2. Models E and F obtain better performance than the baseline model by introducing LCAB and GCAB alone, respectively. This highlights the enhancement in model performance resulting from the incorporation of CNN branches within the Transformer network, underscoring the synergy between the two components. Notably when both LCAB and GCAB are integrated into the network akin to the configuration in model MAFT, the resultant model G exhibits further improved performance, surpassing the baseline model B by achieving enhancements of 0.18 dB and 0.002, respectively. This also suggests that both stronger high frequency feature extraction ability and a larger receptive field are important for Transformer. To visually observe the effect of the CNN branch on the network’s ability to activate pixels, we performed LAM analysis on models E, F, and G, respectively, and the results are shown in Fig. 8. The results show that when either LCAB (model E) or GCAB (model F) are introduced individually, the activation range of pixels is extended to different extents compared to model B. However, including both LCAB and GCAB (model G) activates more pixels and yields superior reconstruction outcomes. We also use the standard MAFT model as model H. The combination of HFE and LCAB, which both aim to extract high frequency information, could result in better reconstruction performance. It is important to note that models E, F and G integrate the output features of the Transformer branch with the CNN branch through T-CFFM.

**Table 2:** Ablation studies on LCAB and GCAB, the metrics PSNR and SSIM are calculated on the Urban100 dataset with a scaling factor of 4.

Model	Model B	Model E	Model F	Model G	Model H
LCAB	$\times$	$\surd$	$\times$	$\surd$	$\surd$
GCAB	$\times$	$\times$	$\surd$	$\surd$	$\surd$
HFE	$\times$	$\times$	$\times$	$\times$	$\surd$
PSNR/SSIM	27.80/0.8351	27.91/0.8362	**27.95/0.8366**	*27.98/0.8371*	*28.06/0.8376*
Parameters(M)	9.87	13.48	14.65	14.07	14.07

The best and second best results are marked in italics and bold, respectively.

#### Effectiveness of window size

To explore the effect of different window sizes on the MAFT reconstruction performance, we set the window sizes of W-MSA and GPHA to 4, 8 and 16 successively for training and show quantitative results with different window sizes for × 4 SR on the five benchmark datasets. The results are shown in Table 3. It is clear that the reconstruction performance of MAFT improves as the window size increases. Compared to the network with the 4 × 4 window size, the network with the 8 × 8 window size achieves a performance Improvement of 0.08 dB, 0.07 dB, 0.04 dB, 0.17 dB and 0.23 dB on the five benchmark datasets, while the network performance is further improved by using the 16 × 16 window size. The Diffusion Index (DI) is employed to illustrate the pixel range of the input images utilized by various models during the reconstruction. A higher DI value signifies a broader range of activated pixels within the network. Using 20 random images from the Urban100 dataset as an example, we compare the distribution of activated pixels in the reconstruction process with different window sizes. As shown in Table 4, increasing the window size leads to a continuous increase in the DI value. This is because a larger window size provides the Transformer network with a greater receptive field, thereby enhancing the network's utilization of pixels. After comprehensive consideration, we ultimately set the window size in the standard MAFT to 16.

**Table 3:** Quantitative results of MAFT with different window sizes for × 4 SR.

Window size	Set5	Set14	BSD100	Urban100	Manga109
(4, 4)	32.94	29.14	27.94	27.65	32.24
(8, 8)	**33.02**	**29.21**	**27.98**	**27.82**	**32.47**
(16, 16)	*33.09*	*29.29*	*28.03*	*28.06*	*32.55*

PSNR used as evaluation metric. The best and second best results are marked in italics and bold, respectively.

**Table 4:** Comparison of the average diffusion index (DI) among different window sizes.

Window size	(4, 4)	(8, 8)	(16, 16)
DI	20.57	22.03	23.86

### Comparison experiments

In order to verify the effectiveness of the proposed MAFT, we compare MAFT with 14 advanced SR methods, which include: SRCNN^7^, EDSR^10^, DBPN^54^, RDN^32^, RCAN^13^, SAN^22^, IGNN^55^, CSNLN^56^, HAN^38^, DRLN^57^, SwinIR ^18^, DLSN^58^, CAT-A^21^ and HAT^59^. The comparison results are classified into several groups according to the upscaling factor.

#### Quantitative evaluation analyses

Table 5 shows the quantitative comparison results of our MAFT and 14 state-of-the-art SR methods under different scale factors. This table shows that our proposed MAFT outperforms other state-of-the-art models on almost all benchmark datasets with scale factors. For example, when the × 4 scale factor is taken as an example and PSNR is used as the evaluation metrics, compared with CAT-A, the reconstruction performance of MAFT designed by us on Set5, Set14, BSD100, Urban100, and Manga109 is improved by 0.03 dB, 0.08 dB, 0.06 dB, 0.19 dB and 0.18 dB, respectively. While comparing with HAT, MAFT achieves performance improvements of 0.07 dB, 0.06 dB, 0.05 dB, 0.11 dB and 0.09 dB on the five baseline datasets. To better demonstrate the superiority of our designed MAFT, we also compared the computational complexity of different SR algorithms at $\times 4$ scale factor. The results are shown in Table 6. Compared to CAT-A, MAFT has 15.24% decrease in parameters and 28.19% decrease in FLOPs. And compared to HAT, MAFT has 32.55% decrease in parameters and 38.01% decrease in FLOPs. This is largely attributed to the effectiveness of sparse attention, which allows MAFT to acquire more global information by requiring fewer Transformer blocks.

**Table 5:** Quantitative results on the SISR benchmark dataset, the best and second best results are annotated in italics and bold, respectively.

Method	Scale	Set5^47^		Set14^48^		BSD100^49^		Urban100^50^		Manga109^51^	
		PSNR SSIM		PSNR SSIM		PSNR SSIM		PSNR SSIM		PSNR SSIM	
Bicubic	× 2	33.66	0.9299	30.24	0.8688	29.56	0.8431	26.88	0.8403	30.80	0.9339
SRCNN^7^	× 2	36.66	0.9542	32.45	0.9067	31.36	0.8879	29.50	0.8946	35.60	0.9663
EDSR^10^	× 2	38.11	0.9602	33.92	0.9195	33.32	0.9013	32.93	0.9351	39.10	0.9773
DBPN^54^	× 2	38.09	0.9600	33.85	0.9190	32.27	0.9000	32.55	0.9324	38.89	0.9775
RDN^32^	× 2	38.24	0.9614	34.01	0.9212	32.34	0.9017	32.89	0.9353	39.18	0.9780
RCAN^13^	× 2	38.27	0.9614	34.11	0.9216	32.41	0.9026	33.34	0.9384	39.43	0.9786
SAN^22^	× 2	38.31	0.9620	34.07	0.9213	32.42	0.9028	33.10	0.9370	39.32	0.9792
IGNN^55^	× 2	38.24	0.9613	34.12	0.9217	32.41	0.9025	33.23	0.9383	39.35	0.9786
CSNLN^56^	× 2	38.28	0.9616	34.12	0.9223	32.40	0.9024	33.25	0.9386	39.37	0.9785
HAN^38^	× 2	38.27	0.9614	34.16	0.9217	32.41	0.9027	33.35	0.9385	39.46	0.9785
DRLN^57^	× 2	38.27	0.9616	34.28	0.9231	32.44	0.9028	33.37	0.9390	39.58	0.9786
SwinIR^18^	× 2	38.42	0.9623	34.46	0.9250	32.53	0.9041	33.81	0.9427	39.92	0.9797
DLSN^58^	× 2	38.49	0.9624	34.51	0.9251	32.53	0.9042	33.98	0.9432	39.89	0.9797
CAT-A^21^	× 2	38.51	0.9626	34.78	0.9265	32.59	0.9047	34.26	0.9440	40.10	0.9805
HAT^59^	× 2	**38.63**	**0.9630**	*34.86*	**0.9274**	**32.62**	*0.9053*	**34.45**	**0.9466**	**40.26**	**0.9809**
MAFT (ours)	× 2	*38.66*	*0.9632*	**34.84**	*0.9278*	*32.65*	**0.9051**	*34.52*	*0.9478*	*40.31*	*0.9816*
Bicubic	× 3	30.39	0.8682	27.55	0.7742	27.21	0.7385	24.46	0.7349	26.95	0.8556
SRCNN^7^	× 3	32.75	0.9090	29.30	0.8215	28.41	0.7863	26.24	0.7989	30.48	0.9117
EDSR^10^	× 3	34.65	0.9280	30.52	0.8462	29.25	0.8093	28.80	0.8653	34.17	0.9476
RDN^32^	× 3	34.71	0.9296	30.57	0.8468	29.26	0.8093	28.80	0.8653	34.13	0.9484
RCAN^13^	× 3	34.74	0.9299	30.64	0.8481	29.32	0.8111	29.08	0.8702	34.43	0.9484
SAN^22^	× 3	34.75	0.9300	30.59	0.8476	29.33	0.8112	28.93	0.8671	34.30	0.9494
IGNN^55^	× 3	34.72	0.9298	30.66	0.8484	29.31	0.8105	29.03	0.8696	34.39	0.9496
CSNLN^56^	× 3	34.74	0.9300	30.66	0.8482	29.33	0.8105	29.13	0.8712	34.45	0.9502
HAN^38^	× 3	34.75	0.9299	30.67	0.8483	29.32	0.8110	29.10	0.8705	34.48	0.9500
DRLN^57^	× 3	34.78	0.9303	30.73	0.8488	29.36	0.8117	29.21	0.8722	34.71	0.9509
SwinIR^18^	× 3	34.97	0.9318	30.93	0.8534	29.46	0.8145	29.75	0.8826	35.12	0.9537
DLSN^58^	× 3	35.02	0.9315	30.90	0.8521	29.47	0.8145	29.77	0.8805	35.20	0.9535
CAT-A^21^	× 3	35.06	0.9326	31.04	0.8538	29.52	0.8160	30.12	0.8862	35.38	0.9546
HAT^59^	× 3	**35.07**	**0.9329**	**31.08**	*0.8555*	**29.54**	**0.8167**	**30.23**	**0.8896**	**35.53**	**0.9552**
MAFT (ours)	× 3	*35.12*	*0.9333*	*31.13*	**0.8549**	*29.58*	*0.8173*	*30.35*	*0.8915*	*35.64*	*0.9568*
Bicubic	× 4	28.42	0.8104	26.00	0.7027	25.96	0.6675	23.14	0.6577	24.89	0.7866
SRCNN^7^	× 4	30.48	0.8628	27.50	0.7513	26.90	0.7101	24.52	0.7221	27.58	0.8555
EDSR^10^	× 4	32.46	0.8968	28.80	0.7876	27.71	0.7420	26.64	0.8033	31.02	0.9148
DBPN^54^	× 4	32.47	0.8980	28.82	0.7860	27.72	0.7400	26.38	0.7946	30.91	0.9137
RDN^32^	× 4	32.47	0.8990	28.81	0.7871	27.72	0.7419	26.61	0.8028	31.00	0.9151
RCAN^13^	× 4	32.62	0.9001	28.86	0.7888	27.76	0.7435	26.82	0.8087	31.21	0.9172
SAN^22^	× 4	32.64	0.9003	28.92	0.7888	27.78	0.7436	26.79	0.8068	31.18	0.9169
IGNN^55^	× 4	32.57	0.8998	28.85	0.7891	27.77	0.7434	26.84	0.8090	31.28	0.9182
CSNLN^56^	× 4	32.68	0.9004	28.95	0.7888	27.80	0.7439	27.22	0.8168	31.43	0.9201
HAN^38^	× 4	32.64	0.9002	28.90	0.7890	27.80	0.7442	26.85	0.8094	31.42	0.9177
DRLN^57^	× 4	32.63	0.9002	28.94	0.7900	27.83	0.7444	26.98	0.8119	31.54	0.9196
SwinIR^18^	× 4	32.92	0.9044	29.09	0.7950	27.92	0.7489	27.45	0.8254	32.03	0.9260
DLSN^58^	× 4	32.95	0.9026	29.14	0.7938	27.92	0.7483	27.49	0.8235	32.10	0.9252
CAT-A^21^	× 4	**33.08**	0.9052	29.18	0.7960	27.99	0.7510	27.89	0.8339	32.39	0.9285
HAT^59^	× 4	33.04	**0.9056**	**29.23**	**0.7973**	**28.00**	**0.7517**	**27.97**	**0.8368**	**32.48**	**0.9292**
MAFT (ours)	× 4	*33.11*	*0.9061*	*29.29*	*0.7978*	*28.05*	*0.7520*	*28.08*	*0.8376*	*32.57*	*0.9297*

**Table 6:** Model complexity comparisons ($\times$ 4).

Method	EDSR	RCAN	SwinIR	CAT-A	HAT	MAFT
Params (M)	43.09	15.59	11.90	16.60	20.86	14.07
FLOPs (G)	823.34	261.01	215.32	360.67	417.81	258.98
Urban100	26.64	26.82	27.45	27.89	27.97	28.08
Manga109	31.02	31.22	32.03	32.39	32.48	32.57

Params (M), FLOPs (G) and PSNR (dB) on Urban100 and Manga109 datasets are reported.

#### Qualitative evaluation analyses

**Figure 9:**
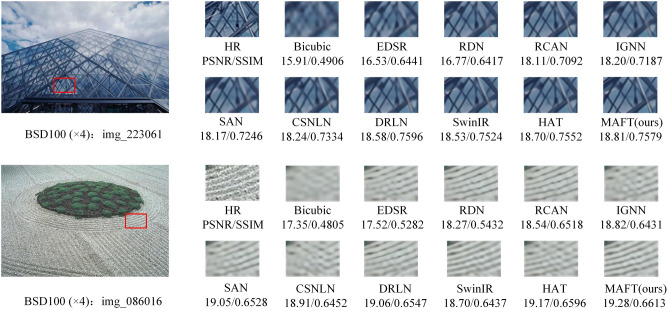
Visual comparison of image SR ($\times$ 4) on BSD100 dataset.

**Figure 10:**
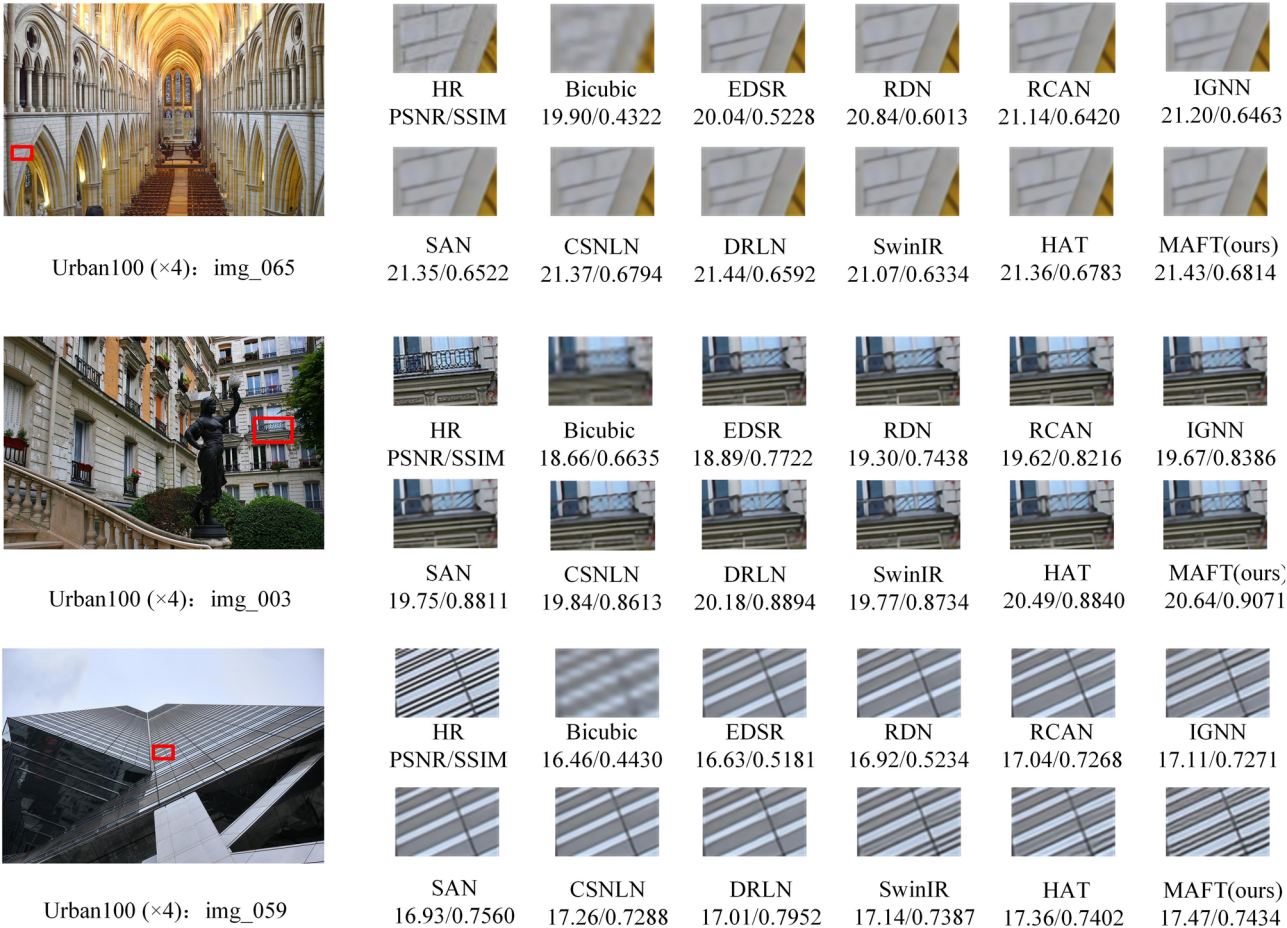
Visual comparison of image SR ($\times$ 4) on Urban100 dataset.

**Figure 11:**
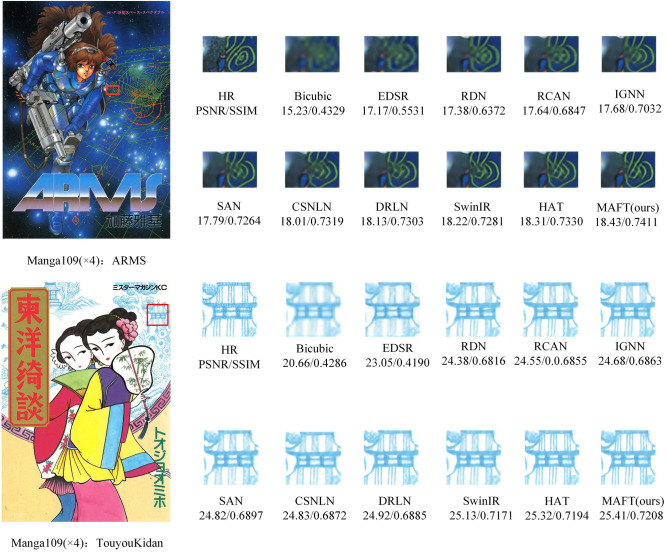
Visual comparison of image SR ($\times$ 4) on Manga109 dataset.

The qualitative comparison was conducted by implementing visualization operations on the proposed MAFT and eight other state-of-the-art (SOTA) methods. Figures 9, 10 and 11 display the visual effects of super-resolution reconstruction achieved by different networks on the BSD100, Urban100, and Manga109 datasets, all with a × 4 scale factor. Notably, the figures illustrate that early super-resolution networks such as EDSR produce images with severe blurring artifacts and a loss of the main image structure. Conversely, DRLN, SwinIR and HAT demonstrate the ability to recover the main contour structure and restore the texture details of the image to a certain extent. Nevertheless, the images reconstructed by these methods still exhibit varying degrees of missing information. In contrast, our proposed MAFT stands out by effectively extracting and reconstructing clearer high-frequency details and texture edges. The reconstruction information provided by MAFT is richer, resulting in more reference and accurate reconstructed images.

## Conclusion

In this paper, we propose the Multi-Attention Fusion Transformer (MAFT), a new image super-resolution reconstruction network based on Transformer. MAFT aims to achieve more satisfactory reconstruction results by increasing the pixel utilization of input features during image reconstruction. In MAFT, we design a new attention module, Global Pixel Hybrid Attention (GPHA), to spatially reorganize global pixel information in the feature map using the Shuffle operation, which effectively enhances the information interaction between different windows. To address GPHA's shortcomings in high-frequency feature extraction capabilities, we design a High-frequency Feature Enhanced (HFE) module, which improve network reconstruction performance without adding computational cost. Additionally, we introduce two CNN-based attention branches connected in parallel with the Transformer branches. This Transformer-CNN parallel connection structure enhances network modeling capabilities, expands pixel utilization range, and ultimately achieves excellent reconstruction performance by leveraging the complementary strengths of both branches. Extensive experiments on multiple datasets demonstrate that the proposed method achieves comparable performance to the current state-of-the-art SR methods while using fewer parameters. However, compared to lightweight networks such as ELAN, MAFT is still a large-scale network with a significant number of parameters due to its complex structure. Therefore, in future work, we will focus on improving the efficiency of MAFT. Furthermore, we will also explore the potential applications of MAFT in image restoration areas such as image denoising and image deblurring.

## Data Availability

The raw data utilized in this study are available upon request to the corresponding author.
